# A Narrative Review of the Role of Blood Biomarkers in the Risk Prediction of Cardiovascular Diseases

**DOI:** 10.7759/cureus.74899

**Published:** 2024-12-01

**Authors:** Lavanya Garady, Ashok Soota, Yogesh Shouche, Komal Prasad Chandrachari, Srikanth K V, Prasan Shankar, Sanketh V Sharma, Kavyashree C, Shrutika Munnyal, Ahalya Gopi, Azad Devyani

**Affiliations:** 1 Public Health Sciences, Scientific Knowledge for Ageing and Neurological Ailments (SKAN) Research Trust, Bengaluru, IND; 2 Information Technology, Scientific Knowledge for Ageing and Neurological Ailments (SKAN) Research Trust, Bengaluru, IND; 3 Microbiology, Scientific Knowledge for Ageing and Neurological Ailments (SKAN) Research Trust, Bengaluru, IND; 4 Neurosurgery, Narayana Institute of Neurosciences/Mazumdar Shaw Medical Center, Bengaluru, IND; 5 Cardiology, Narayana Institute of Cardiac Sciences, Bengaluru, IND; 6 Ayurvedic Medicine, Institute of Ayurveda and Integrative Medicine (I-AIM) Healthcare Center, The University of Trans-Disciplinary Health Sciences and Technology, Bengaluru, IND; 7 Ayurvedic Medicine, The University of Trans-Disciplinary Health Sciences and Technology, Bengaluru, IND

**Keywords:** blood biomarkers, cardiovascular disease (cvd), myocardial infarction, risk prediction, stroke

## Abstract

Cardiovascular disease (CVD) is a global health crisis and a leading cause of morbidities and mortalities. Biomarkers whose evaluation would allow the detection of CVD at an early stage of development are actively sought. Biomarkers are objectively measured as indicators of health, disease, or response to an exposure or intervention, including therapeutic interventions. Hence, this review aims to identify biomarkers that can help predict CVD risk in the healthy population. This helps with risk prediction and is crucial for advancing preventive cardiology and improving clinical outcomes in a wide range of patient populations. Biomarkers such as atherogenic lipoproteins, fibrinogen, homocysteine, and thyroid-stimulating hormone (TSH) have been linked to CVD risk factors, including dyslipidemia, hypertension, diabetes, and obesity. When combined with conventional biomarkers, inflammatory markers such as C-reactive protein (CRP) can enhance risk prediction. However, biomarkers such as high-sensitivity troponin T (hsTnT) and N-terminal proBNP (NT-proBNP) are widely used as diagnostic biomarkers for heart failure (HF) and cardiac dysfunction, as they are released only after one to two hours of cardiovascular event occurrence. Myeloperoxidase (MPO) and procalcitonin (PCT) have developed into promising new biomarkers for the early detection of systemic bacterial infections as inflammatory markers, which are better diagnostic tools than screening. Combining biomarkers can improve test accuracy, but the best combinations for diagnosis or prognosis must be identified.

## Introduction and background

Cardiovascular disease (CVD) refers to a group of disorders affecting the heart and blood vessels, including atherosclerotic peripheral arterial disease (PAD) and coronary artery disease (CAD) [1]. CVD stands as a global health crisis, being the leading cause of morbidity and mortality in this world of aging population [2]. In 2019, statistics unveiled a striking figure of 523 million people impacted by CVD, resulting in 18.6 million fatalities attributed to these ailments [1]. Conventional risk factors such as hypertension, diabetes mellitus, smoking, and high lipid levels have played a pivotal role in shaping risk prediction models and advancing treatment methods in conjunction with biomarkers [2].

Biomarkers are "characteristics that are objectively measured as indicators of health, disease, or a response to an exposure or intervention, including therapeutic interventions" [2,3]. Biomarkers can be categorized as diagnostic, predictive, and prognostic markers. Diagnostic biomarkers are used to detect or confirm the presence of a disease or a condition. A prognostic biomarker provides information about the progression of a disease in an untreated individual or one getting routine treatment. A predictive biomarker aids in the identification of individuals who are most likely to benefit from a specific therapy or distinguishes those who are suitable for targeted therapies. On the other hand, pharmacodynamic biomarkers assess the impact of a drug on the disease itself. Essentially, they reflect how a target organism changes in response to both the disease and its treatment. Over the past 30 years, advances in CVD biomarker research and innovations resulted in more sensitive screening techniques, increased focus on early detection and diagnosis, and better treatments that have improved clinical outcomes in the community [3]. Biomarkers are considered valuable when they fulfill certain criteria, such as (a) accuracy or the capacity to recognize people who are at risk, (b) reliability and the consistency of outcomes upon repeating, and (c) the therapeutic effect of early intervention [2].

Assessing cardiovascular risk in high-risk asymptomatic individuals is now a common practice in preventive medicine. Predictive tools and risk scores, developed from comprehensive cohort studies and randomized trials, help to pinpoint those vulnerable to CVD [4]. However, traditional risk-assessment models, such as the Framingham risk score, may no longer fully reflect current health patterns due to significant changes across generations [5].

This comprehensive search aims to uncover novel biomarkers that not only complement existing risk-assessment models but also offer additional insights into cardiovascular health.

Methodology 

An in-depth literature review was conducted to improve risk prediction tools, utilizing keywords such as "risk prediction", "biomarker", "lab tests", and CVD-related terms such as "acute coronary syndrome", "coronary artery disease", "myocardial infarction", "heart failure", and "stroke", on PubMed and Google Scholar.

Inclusion and Exclusion Criteria

**Figure 1:**
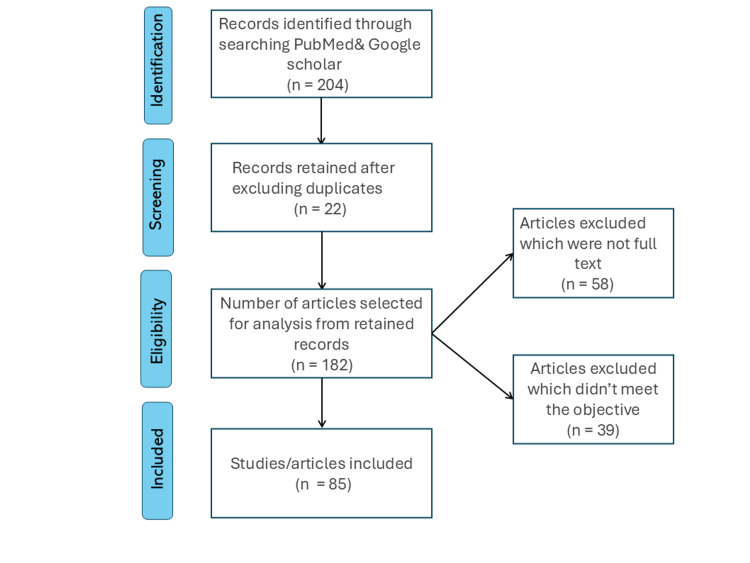
A PRISMA schematic representation of the article search PRISMA: Preferred Reporting Items for Systematic Reviews and Meta-Analyses

This review included studies published exclusively in English, without any specific geographic location, covering the period from 2000 to 2024. Additionally, only free full-text peer-reviewed journal articles were included. Studies are excluded if they do not contain the required biomarker information (Figure 1).

## Review

**Figure 2:**
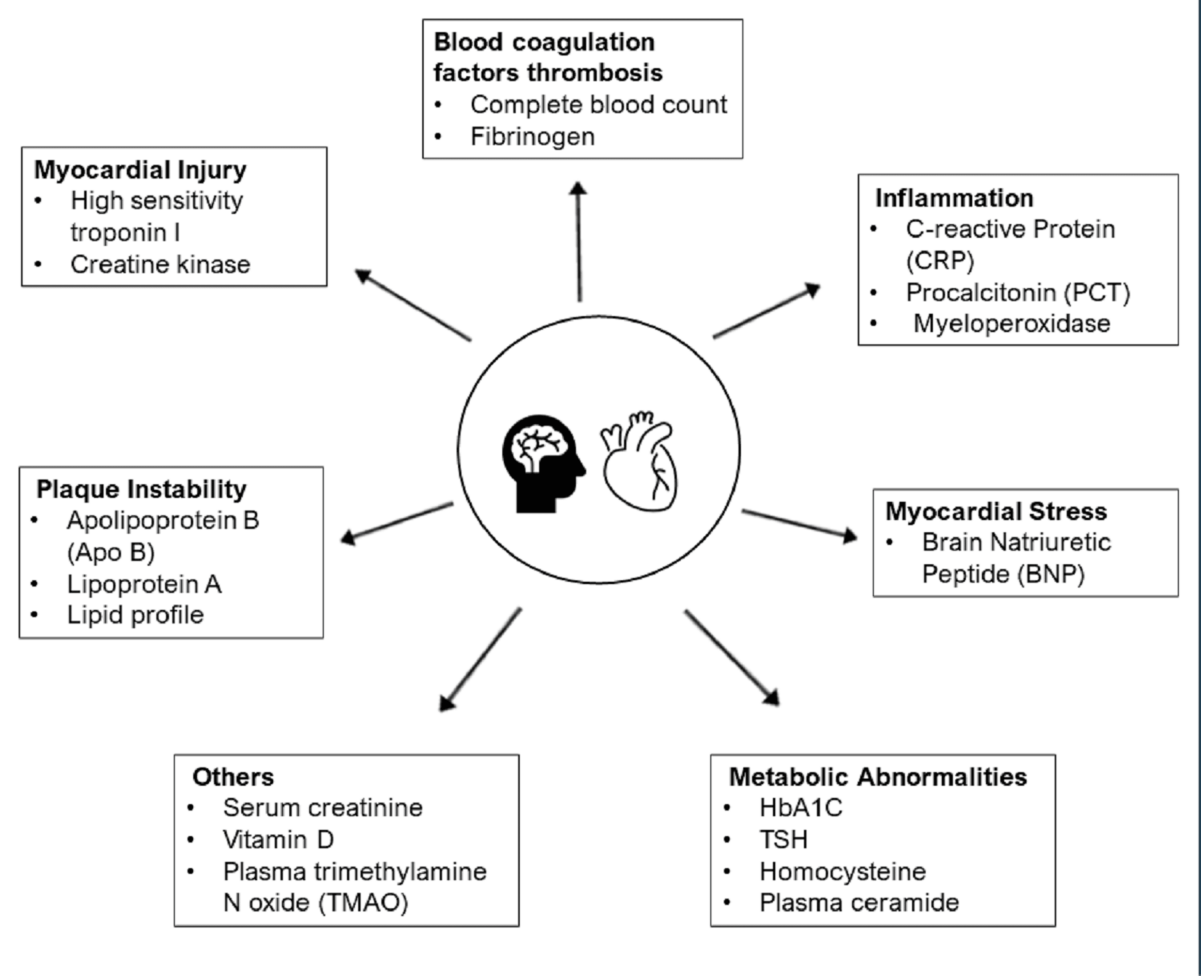
Causes of cardiovascular disease (CVD) and their blood biomarkers TSH: thyroid-stimulating hormone; HbA1c: glycated hemoglobin A1c Image credits: Kavyashree C

Cardiovascular biomarkers are classified according to their pathophysiological processes, such as (1) myocardial injury, (2) myocardial stress, (3) inflammation, (4) blood coagulation factors (platelet activation), (5) plaque instability, and (6) metabolic abnormalities (Figure 2) [2,6].

Biomarkers indicating metabolic abnormalities

Glycated Hemoglobin A1c (HbA1c)

HbA1c is a blood test that measures average blood glucose concentrations from the past two to three months [7]. High HbA1c levels indicate a higher occurrence of carotid arterial plaque in both pre-diabetic and diabetic patients. Arterial plaque leads to increased development of coronary heart disease (CHD) [8]. A study by Ceriello et al. found that individuals with a mean HbA1c level of 53 mmol/mol had a higher risk of myocardial infarction, stroke, and mortality compared to those with a lower HbA1c level [9]. According to Khaw et al., a 1% increase in HbA1c was linked to a 21% increase in cardiovascular risk in both men and women [10]. In chronic hyperglycemic individuals, the chances of CVD risk are higher; however, maintaining HbA1c levels less than 70 mmol/mol (8.6%) may reduce CVD risk in diabetes patients. However, findings regarding the association between abnormal HbA1c levels and cardiovascular complications are inconsistent among healthy populations [11,12].

Thyroid-Stimulating Hormone (TSH)

TSH plays a crucial role in regulating metabolism, influencing thermogenesis, and controlling energy expenditure [13]. A population-based study indicates that hyperlipidemia and vascular inflammation are caused by hypothyroidism (high TSH value). Hyperlipidemia leads to systolic hypertension, atrial fibrillation, and hypercoagulability. The effects of dyslipidemia directly contribute to the development of atherosclerotic CVD [14,15]. Serum TSH levels higher than 10 mIU/L among young populations with subclinical hypothyroidism increase the risk of CHD and mortality [14]. Therefore, participants with high TSH levels have a significantly higher risk of CVD mortality. TSH levels play a crucial role in identifying the high-risk population in the community [16,17].

Homocysteine

Homocysteine is an amino acid that is a byproduct of meat and dairy products. It interacts with vitamins B12 and B6 and folate to form the proteins needed by the body. Normally, very little homocysteine stays in a healthy individual's blood. Homocysteine plays a crucial role in endothelial clot formation by inhibiting protein C and heparin sulfate, which in turn increases blood viscosity. Furthermore, serum homocysteine interacts with low-density lipoprotein (LDL) to form LDL-homocysteine thiolactone, which forms atherosclerotic plaques and thrombosis in arteries. The risk of atherosclerosis increases with age due to an increase in homocysteine levels [18,19]. A study by Lühmann et al. showed that lowering homocysteine levels with folate or vitamin B does not reduce the risk of cardiac events in high-risk populations. Homocysteine might not be a strong predictor individually, but in combination with standard risk factors, it can give a stronger predictive value in healthy individuals [20].

Plasma Ceramides

Plasma ceramides are fatty acids present in all tissues and blood that play a role in cellular signaling. It accumulates when there is inflammation, dyslipidemia, or metabolic malfunction. Elevated ceramide levels modify cell membrane permeability, leading to leaky blood vessels and plaque accumulation. In addition, ceramides form 30% of circulating LDL-cholesterol, which plays a pivotal role in atherosclerosis [21,22]. Mishra et al. reported that increased plasma ceramides correlate with a 9% rise in the risk of impaired left ventricular function. The impaired left ventricle reduces the ejection fraction, which increases CVD mortality. Nevertheless, plasma ceramides could serve as a stand-alone predictive biomarker for high carotid intima-media thickness (CIMT), indicating their potential in identifying subclinical atherosclerosis [23].

Biomarkers indicating plaque instability

Lipid Profile

Lipoproteins are particles made of protein and fats, categorized into atherogenic and anti-atherogenic. Atherogenic factors such as LDL and very low-density lipids (VLDL) tend to promote atherosclerosis. Anti-atherogenic, such as high-density lipids (HDL), inhibits atherosclerosis. Increased atherogenic levels increase macrophage absorption of cholesterol, which in turn causes inflammation and plaque formation. This mechanism has made dyslipidemia the major risk factor for CVD [24]. Notably, non-HDL, which consists of atherogenic particles, is emerging as a stronger predictor of CVD risk irrespective of triglyceride (TG) levels [25]. Tanabe et al. suggested that non-HDL cholesterol levels are significant indicators for assessing the risks. The findings suggest that considering non-HDL cholesterol, which includes various atherogenic lipoproteins, can enhance the accuracy of cardiovascular risk prediction compared to total cholesterol alone [26].

TG play a pivotal role in lipid metabolism due to their association with atherogenic particles, which increase the chance of plaque formation. Increased TG causes pancreatitis and other conditions, which are linked to increased atherosclerotic risk [27]. However, some studies suggested that TG is not an independent risk factor for measuring CVD risk prediction [28]. Moreover, the Atherosclerosis Risk in Communities (ARIC) study revealed strong associations of total cholesterol, LDL, and TG with increased CVD risk, while HDL is linked to decreased risk. Evidence from the studies suggested that reduced LDL level by every mmol/L will reduce the risk of CVD by 40% [29], and a similar study mentioned that a 12% increase in CVD risk is associated with every 10 mg/dL rise in LDL [30]. Therefore, total cholesterol/HDL and LDL/HDL ratios are better predictors of CVD risk instead of focusing solely on LDL-cholesterol levels [31]. Additionally, a recent study by Gentile et al. revealed an independent association between VLDL and CIMT [32], where a positive association was observed between VLDL and vessel thickness, arterial stiffening, and loss of elasticity, which account for major cardiovascular risk factors [33]. Therefore, including whole lipid particles will increase accuracy in predicting CVD risk better than using only LDL and HDL.

Apolipoprotein B (ApoB)

ApoB is a protein component of LDL, which measures the number of atherogenic particles [34], including LDL. It is an accurate predictor of CVD events [29,35], and combining ApoB with traditional lipids significantly improves long-term CVD risk assessment [36]. Studies show that increased ApoB particles lead to the development of ischemic heart disease, myocardial infarction, and other CVD events [34,37]. Su et al. demonstrated that adding ApoB information to LDL and HDL measurements does not significantly enhance CVD risk prediction based on China's atherosclerotic CVD (ASCVD) risk score. Overall, ApoB's addition improves CVD risk assessment [38].

Lipoprotein A (Lp A)

Lp A binds to oxidized phospholipids to transport cholesterol particles. It also plays a critical role in macrophage foam cell formation, which in turn aids in the development of thrombosis and atherosclerosis [39]. This process ultimately results in the buildup of plaques, leading to atherosclerosis. The accumulated lipid plaques cause aortic valve stenosis, leading to valve calcification [40]. A study by Mohammadi-Shemirani et al. showed that a 50 mg/dL increase in Lp A will increase the risk of atrial fibrillation by 3% [41] and the risk of CVD by 20% [42]. Therefore, Lp A has been found to be a significant CVD risk prediction, particularly among low-risk individuals [43].

Biomarkers indicating inflammation

C-reactive Protein (CRP)

CRP levels increase in response to tissue damage caused by trauma, infection, malignancy, or chronic inflammatory conditions [44]. This indicates the linear association between CRP level and the risk of CVD, stroke, and CHD [45], making CRP a better inflammatory marker for predicting CVD events. Specifically, high CRP levels in acute myocardial infarction indicate inflammation and thrombosis in the infarcted myocardium [46,47]. In contrast, a study by Han et al. showed that the use of CRP in preventive care is still uncertain [48] because the levels can be influenced by multiple factors, such as chronic inflammatory diseases, lifestyle, and obesity [44]. Due to these influences, CRP is not used as a sole marker in asymptomatic individuals. Instead, CRP is often considered alongside other risk factors and diagnostic tools to provide a more comprehensive CVD assessment [49].

Myeloperoxidase (MPO)

MPO is a protein released by circulating leukocytes during CVD events, and upon oxidation, macrophages enriched in MPO produce hypochlorous acid (HOCl) [50]. This chemical contributes to increased inflammation and plaque formation with thinner fibrous caps, which is the leading cause of coronary thrombosis, infarction, and sudden cardiac mortality events between 30 days and six months [51]. The elevated MPO levels in coronary circulation suggest localized tissue injury due to pathophysiological processes of cardiac failure. Therefore, MPO can be used as a diagnostic and monitoring marker but not in prediction [52,53].

Procalcitonin (PCT)

PCT is a protein released by macrophages associated with sepsis, progressive atherosclerosis, obesity, and insulin resistance. Elevated PCT indicates the severity of bacterial infection. Patients with severe myocardial damage post-infarction showed increased PCT concentrations, leading to increased mortality within 48 hours to six months. PCT enhances outcome and prognosis information but is not as accurate as CRP in predicting outcomes [54]. The serum PCT levels were significantly higher among the CVD high-risk populations than those with low to intermittent-risk populations [54,55]. High PCT values have a twofold increased risk of cardiovascular death. PCT has a lesser association with the prediction of stroke development than myocardial infarction [56].

Blood coagulation factors and thrombosis

Complete Blood Count (RBC, WBC, Platelet, and Hematocrit)

A complete blood count (CBC) is a laboratory test that measures the levels of red blood cells (RBC), white blood cells (WBC), platelets, hemoglobin, and hematocrit in an individual's blood. Most studies reported a consistent association between elevated RBC, WBC, hematocrit, and CVD. Similarly, a higher RBC count and hematocrit levels contribute to increased blood thickness and increased platelet clumping by releasing adenosine diphosphate. The thickened blood and platelet clumps have an increased chance of atherosclerotic plaque formation, increasing CVD risk [57]. The above-mentioned mechanism was also reported to cause vascular smooth muscle dysfunction and abnormal vascular structure [58]. Moreover, higher RBC and hemoglobin levels are associated with increased risk factor development, such as hypertension, diabetes, dyslipidemia, and obesity, which potentially augments plaque formation in coronary arteries. Consequently, platelets are essential in clotting and inflammatory mechanisms by encouraging the attachment of inflammatory cells, such as neutrophils and monocytes, causing plaque and thrombosis formation. The elevated platelets (thrombocythemia) increase the frequency of thrombosis formation [59].

Neutrophils and monocytes, a type of WBC, act as the first line of defense in the immune mechanism. A prospective cohort study conducted across Europe, which aims to study the etiology of chronic diseases, revealed that elevated WBC counts, particularly among active smokers, were linked to increased CVD risk, with a stronger effect on stroke than on CHD. Despite the not-so-significant relation between individual WBC subtype associations with CVD, when total WBC count is taken into consideration, it revealed a strong association between WBC count and CVD risk [60]. WBC count was found to predict future CVD and mortality in patients with or at high risk, independently of conventional risk factors. The greatest predictive ability was provided by high neutrophil (N) or low lymphocyte (L) counts [61]. The N/L ratio measures the balance of inflammation and immunity in the body. The higher the N/L ratio, the higher the chance of CVD. These findings have important implications for CVD risk assessment [62].

Fibrinogen

Fibrinogen is a glycoprotein, which is also known as coagulation factor I. Fibrinogen plays a crucial role in blood clotting. Fibrinogen concentration is affected by factors that regulate synthesis and genetic factors. It has a half-life of three to five days [1,63]. Increased fibrinogen leads to the formation of thrombosis, which blocks the blood vessels and reduces blood supply, causing CVD. According to the Framingham trial, greater frequency of CVD events was associated with higher fibrinogen plasma concentrations [64]. Furthermore, a study by Surma et al. reported that the individuals who experienced a myocardial infarction event showed higher plasma fibrinogen concentrations (≥343 mg/dL) than healthy individuals [1]. A study by Maresca et al. showed that arterial thrombosis occurs irrespective of fibrinogen levels [65]. Overall, these findings highlight the significance of fibrinogen as a predictor of CVD risk and its potential role in refining risk-assessment strategies [66].

Biomarkers indicating myocardial injury

Creatine Kinase (CK)

CK biomarkers are known to be used to diagnose muscle disorders. CK is an enzyme that is generally found in muscles of the skeletal, heart, and brain [67]. When heart muscles are damaged due to atherosclerosis, CK is released into the bloodstream. This makes it inadequate for early diagnosis of myocardial infarction within six hours of the event [68]. A study by Wu et al. noted that CVD mortality is higher among the groups with high CK values than the low CK group [69]. Therefore, CK is a better diagnostic marker than predicting the risk of CVD.

Cardiac Troponin

Cardiac troponin I (cTnI) is a protein that is released into the bloodstream from myocardial cells when they are permanently damaged due to acute heart muscle injury. cTnI levels are usually elevated four to nine hours after myocardial injury in the bloodstream, peaking at 12-24 hours. Increased cTnI levels may not appear for two to three hours after a myocardial injury due to several physiological processes involved in the release. It takes time for myocyte membranes to break down and release detectable levels of troponins into the blood. This process typically begins within hours of the onset of myocardial injury, making it difficult to predict cardiovascular events [70]. Higher troponin levels indicate more damage to the heart muscles [71] and are associated with CHD, mortality, and heart failure events [72].

In contrast, a study by Huynh [71] and Blankenberg et al. [73] mentioned that high-sensitivity troponin I (hs-TnI) is an advanced diagnostic that aids in detecting low troponin concentrations, enabling earlier and more accurate diagnosis of myocardial infarction. Therefore, the hs-TnI test helps in early diagnosis, prognosis, and potential treatment strategies for CVD.

Biomarkers indicating myocardial stress

N-terminal Prohormone of Brain Natriuretic Peptide (NT-proBNP)

NT-proBNP is a protein-based hormone produced by the ventricles of the heart. It is released in response to cardiac wall stress during heart failure events as a protection mechanism for reducing the workload on the heart and improving its efficiency. The mechanism leads to vasodilation and diuresis to minimize cardiac workload [74]. Increased cardiac muscle damage correlates with higher NT-proBNP levels, which are associated with increased cardiovascular mortality rates [75]. A study by Hussain et al. reported that participants with hypertension and elevated NT-proBNP levels had greater cardiovascular risk compared with those with hypertension but with lower NT-proBNP levels [76]. Pulmonary and renal dysfunction can also increase NT-proBNP levels [74]. Therefore, the NT-proBNP test can predict morbidity and mortality of cardiovascular events, which benefits heart failure screening, identifying people at high risk of heart failure, and helping manage disease progression [75].

Others

Serum Creatinine

Serum creatinine is the marker of kidney function. Elevated creatinine levels and decreased glomerular filtration rate (GFR) are indicators of impaired kidney function. Impaired kidney function is associated with fluid retention and atherosclerotic plaque formation [77]. These increased fluids cause hypertension and increased cardiac afterload. This association suggests that even mild impairment of renal function can contribute to an elevated risk of CVD events and mortality [78]. A study by Chen et al. mentioned that higher creatinine levels are recognized among elderly, diabetic, and hypertensive individuals and those with a history of myocardial infarction or stroke [79]. Therefore, serum creatinine can be used as a marker to assess the high-risk populations for CVD [77].

Vitamin D

Vitamin D, also known as calciferol, is primarily produced in the body by the action of sunlight on the skin, although it may also be taken through food sources and supplements. Vitamin D plays a crucial role in calcium absorption and metabolism, which is essential for bone mineralization [80]. Vitamin D is essential for maintaining endothelial function, which prevents platelet aggregation and controlling inflammation. Vitamin D deficiency (<20 ng/mL) can cause endothelial dysfunction, which is an early stage in the development of atherosclerosis [81] and is linked to a twofold increase in the risk of CVD events [82]. Additionally, vitamin D deficiency was associated with various CVD risk factor development, such as hypertension, diabetes, high body mass index (>30), and elevated triglyceride levels [83]. In contrast, high vitamin D levels lead to irregular heartbeat and atrial fibrillations [83].

Plasma Trimethylamine N-oxide (TMAO)

TMAO is a small colorless amine oxide generated from choline, betaine, and carnitine by gut microbial metabolism, which is rich in many fruits, vegetables, nuts, dairy products, and meat [84]. The elevated levels of TMAO (>6 μM) [85] alter metabolism, which influences bile acid synthesis and cholesterol absorption, causing dysfunction in vascular cells and cardiomyocytes. The elevated TMAO also promotes the accumulation of cholesterol in macrophages, leading to foam cell formation. The dysfunction in vascular cells leads to inflammation and cellular apoptosis, which contributes to the development of atherosclerosis, cardiomyopathy, and heart failure [84,86]. Moreover, in a European study, CVD patients had higher plasma TMAO levels than healthy individuals. The study revealed that gut microbiota-related mechanisms contributed to CVD progression, but the predictive value of TMAO needs further evaluation [86].

Table 1 presents biomarkers and their normal ranges, along with their relevant references.

**Table 1:** Biomarkers and their normal ranges

Sl. No.	Biomarkers	Normal Range	Reference
1	Glycated hemoglobin A1c (HbA1c)	<5.6%	Cavero et al. [7]
2	C-reactive protein (CRP)	<2 mg/L	Ge et al. [3]
3	Thyroid-stimulating hormone (TSH)	0.5–5.0 IU/mL	Souza et al. [13]
4	Hemoglobin	13–18 g/dL (men) and 12–16 g/dL (women)	El Brihi et al. [87]
5	Red blood cell (RBC)	4.6–6.2 million cells/μL	El Brihi et al. [87]
6	White blood cell (WBC)	4500–11000 cells/μL	El Brihi et al. [87]
7	Hematocrit	40%–54%	El Brihi et al. [87]
8	Low-density lipoprotein (LDL)	< 130 mg/dL	Ge et al. [3]
9	Triglycerides	<150 mg/dL	Miller et al. [24], Singh et al. [25]
10	High-density lipoprotein (HDL)	40 mg/dL	Singh et al. [25]
11	High-sensitivity troponin I (hs-TnI)	<14 ng/L	Everett et al. [72]
12	Plasma ceramide	0–6 score	Junqueira et al. [21]
13	Procalcitonin (PCT)	<0.1 ng/mL	Schiopu et al. [56]
14	Myeloperoxidase (MPO)	0–350 μg/L	Baldus et al. [52]
15	NT-pro-brain natriuretic peptide (NT-proBNP)	<125 pg/mL (<=75 age); <450 pg/mL (>75 age)	Doust et al. [75]
16	Creatine kinase	80–200 IU/L	Al-Hadi et al. [68]
17	Apolipoprotein B (Apo B)	63–130 mg/dL	Benn et al. [34]
18	Lipoprotein A	<30 mg/dL or <75 nmol/L	Mohammadi et al. [41]
19	Homocysteine	5–15 μmol/L	Moradi et al. [18]
20	Vitamin D	10–50 ng/mL	Judd et al. [80]
21	Plasma trimethylamine N-oxide (TMAO)	<6 μM	Tang et al. [85]
22	Serum creatinine	0.7–1.3 mg/dL	American Kidney Fund [88]

Limitation

Articles in other languages, such as Mandarin and Spanish, may have been excluded. This language restriction might have led to a "Tower of Babel" bias. Since such articles are easily accessible and understandable, we included them in our narrative review.

## Conclusions

Biomarkers are often used for diagnosis, prognosis, and risk prediction. The extensive literature conducted focuses on identifying biomarkers whose varied levels are useful in identifying high-risk populations and early diagnosis of all aspects of CVD event occurrence. The increased levels of biomarkers such as atherogenic lipoproteins, fibrinogen, homocysteine, and TSH showed a greater association with CVD risk factor development. Risk factors such as dyslipidemia, hypertension, diabetes, and obesity cause atherosclerotic CVD. Inflammatory markers such as CRP will not provide specific risk, but they can enhance risk prediction while adding conventional biomarkers. Biomarkers such as hs-TnI, NT-proBNP, MPO, PCT, CK, and plasma ceramide act as better diagnostic markers than screening, as some proteins are released from cardiac myocytes into the bloodstream only one to two hours following the onset of a cardiovascular event. Although there is evidence that combining biomarkers can improve the accuracy of specific tests, the best combinations for early diagnosis or prognosis must be identified.
